# Editorial

**DOI:** 10.1120/jacmp.v1i1.2649

**Published:** 2000-01-01

The American College of Medical Physics (ACMP) is pleased to present the first issue of the Journal of Applied Clinical Medical Physics (JACMP). The ACMP was formed inpart, “to promote the continuing competence of the practitioners of medical physics” (ACMP Bylaws Article II Objectives). Although dissemination of the practical aspects of new work is very much part of this purpose, the college had no journal in which such work could be presented. A few years ago, a task group was formed to evaluate the possibility of starting a journal of clinical physics. This task group determined that a journal in applied clinical medical physics in its broadest interpretation, including technical, regulatory, managerial, fiscal, and educational concerns, was needed. It also determined that with current technology an all‐electronic journal would be desirable.

In 1998 the Editor‐in‐Chief was selected and an Editorial Advisory Board was appointed to get the new journal started. They also formulated the mission statement of the journal, i.e,. to publish papers that will help clinical medical physicists perform their duties more effectively, efficiently, and for the increased benefit to the patient.

Although numerous journals were on the internet at that time, they were primarily print journals that were posted on the internet. Systems for handling an entirely electronic journal, including submission, review, editing, advertising, etc. were just beginning to be developed. After careful review the Editor‐in‐ Chief and the Advisory Board recommended to the ACMP Board of Chancellors that a contract with the American Institute of Physics (AIP) be executed to produce the journal with ACMP. AIP was in the process of developing suitable computer systems, with some of its member societies, to produce allelectronic journals and the JACMP was the logical next step in this development. After approval by the Board of Chancellors at the 1999 annual meeting, a Manuscript Manager was appointed and goals were set to publish the first issue of the JACMP in early 2000.

It is a pleasure to acknowledge the help and encouragement from many sources and in particular from the personnel at AIP who have gone well beyond the terms of their contract in getting the journal started. Each of you also have a vital role to play in the further development of the journal by publicizing it among your friends and colleagues, submitting manuscripts for consideration to be published, supporting the advertisers, and encouraging other corporations to advertise, and finally by telling us how the journal can be improved.

Welcome to the JACMP.

Peter R. Almond, Ph.D.

Editor‐in‐Chief

